# Complete Genome Sequence of vB_EcoM_112, a T-Even-Type Bacteriophage Specific for *Escherichia coli* O157:H7

**DOI:** 10.1128/genomeA.00393-14

**Published:** 2014-11-13

**Authors:** Brid Coffey, R. Paul Ross, Gary O’Flynn, Orla O’Sullivan, Aidan Casey, Michael Callanan, Aidan Coffey, Olivia McAuliffe

**Affiliations:** a Teagasc Food Research Centre, Moorepark, Fermoy, County Cork, Ireland; b Department of Biological Sciences, Cork Institute of Technology, Bishopstown, Cork, Ireland

## Abstract

Bacteriophage vB_EcoM_112 (formerly e11/2) is an *Escherichia coli* phage with specificity for the O157:H7 serotype. The vB_EcoM_112 genome sequence shares high degrees of similarity with the phage T4 genome sequence.

## GENOME ANNOUNCEMENT

The T4 superfamily is an ideal model system in which to study phage diversity. More than 40 T4-related phages have been sequenced, with many sharing morphological and genetic similarities, but diverging in terms of host range, genome sequence, size, gene positions within the genome, and capsid size (1). T4 relatives appear to have diverged from a common ancestor by changing their ability to infect different host bacteria and by occupying new ecological niches (1–3).

Bacteriophage vB_EcoM_112 (formerly e11/2), a *Myoviridae* member isolated from bovine slurry (4), has shown significant potential as a biocontrol agent for *Escherichia coli* O157:H7 (5, 6). The genome of vB_EcoM_112 was sequenced by Beckman Coulter Genomics, (Meylan Cedex, France) on a 454 GS-FLX NextGen sequencing platform (Roche Diagnostics GmbH). Sequencing yielded a total of 31,993 reads, with an average read length of 585 bp and average coverage of 111. Sequence assembly was performed with Consed assembly package (7), open reading frames (ORFs) identified using GLIMMER 3.02 (8), and resulting gene models were fed into GAMOLA for annotation (9). The automated annotation was manually verified in ARTEMIS (10) using BLASTp. Conserved domain searches were performed using InterProScan (11) and TigrFam (12). Protein Motif analysis was carried out using the Prosite server http://www.expasy.org/prosite.

The vB_EcoM_112 genome consists of 168,470 bp of dsDNA with a G+C content of 35.28%. PCR analysis indicated that the genome can exist as a closed circle. vB_EcoM_112 phage particles contain linear, circularly permuted DNA with terminal redundancy. A total of 270 ORFs were identified on the genome, 222 of which exhibit highest levels of similarity to proteins from T4. Putative functions were assigned to 138 ORFs, including replication, structural proteins, lysis, and DNA packaging. In total, 126 hypothetical proteins were identified, with 61 of these similar to T4 conserved hypothetical proteins and 26 ORFs with similarities to other phage-related proteins. Six annotated ORFs appear to be unique to vB_EcoM_112. A number of key differences distinguish vB_EcoM_112 from T4. The presence of a β-glucosyl-α-glucosyltransferase gene (ORF 21) in vB_EcoM_112 in place of the T4 β-glucosyltransferase gene for the glucosylation of hydroxymethyl dCMP DNA is a feature of T-even phages other than T4 (1, 13). Homing endonucleases are also less prevalent in vB_EcoM_112, which contains nine endonucleases (both free-standing and intron-encoding), compared to T4’s fifteen endonucleases. Significantly, the tail fiber region also distinguishes vB_EcoM_112 from T4 and other T-even phages. The products of ORF 225 and ORF 226 share significant identity to tail proteins from *E. coli* O157:H7-specific phages PPO1 (3) and AR1 (14), but not to corresponding proteins in other T-even phages. These findings may explain the variation in host specificity of these phages. Furthermore, the lack of any features in the genome which may hinder its inclusion in live animal trials or food studies, e.g., toxins, strengthens the potential for development of phage vB_EcoM_112 as a potent biocontrol agent.

### Nucleotide sequence accession number.

The complete genome sequence of *E. coli* phage vB_EcoM_112 has been deposited in GenBank under accession no. KJ668714.
